# The Differential Impact of Neoadjuvant Therapies on the Tumor Microenvironment, Peripheral Biomarkers, and Survival in Pancreatic Cancer: A Retrospective Cohort Study

**DOI:** 10.3390/cancers18101567

**Published:** 2026-05-12

**Authors:** Trevor Silva, Tomoko Yamazaki, John M. Creasy, Jon M. Gerry, Binbin Zheng-Lin, Amar J. Srivastava, Kristina H. Young

**Affiliations:** 1 Providence Cancer Institute, Portland, OR 97213, USA; 2 The Oregon Clinic, Portland, OR 97213, USA

**Keywords:** pancreatic cancer, neoadjuvant therapy, radiation, neutrophil-to-lymphocyte ratio, tumor microenvironment

## Abstract

**Background/Objectives**: The selection of neoadjuvant therapy for patients with non-metastatic pancreatic adenocarcinoma remains challenging. **Methods**: We performed a single-institution, retrospective analysis of 79 patients who underwent resection of their pancreatic adenocarcinoma after receiving neoadjuvant therapy. Clinical and pathologic data were collected. Tumor fibrosis was quantified using Masson’s trichrome staining, tumor-infiltrating lymphocytes (TIL) were evaluated by an AI-based analysis of whole-slide H&E images, and immune cell populations were quantified by multiplex immunohistochemistry. Correlation analyses were performed between neoadjuvant treatment regimen, tumor regression, immune phenotypes, and survival. **Results**: All patients received chemotherapy, 77% FOLFIRINOX and 23% Gemcitabine/nab-paclitaxel (Abraxane). Eighteen percent of patients went on to receive radiation. Tumor regression grade (TRG) correlated with the neoadjuvant regimen. A reduction in tumor markers and the baseline neutrophil-to-lymphocyte ratio (NLR) correlated with overall survival. Among patients with an NLR > 3.3, FOLFIRINOX conferred a survival benefit over Gemcitabine/nab-paclitaxel, and radiation trended towards improved survival. Radiation was associated with increased fibrosis and reduced infiltration of CD8^+^ and regulatory T cells (Tregs). Increased Tregs and PDL1^+^ stromal cells were associated with poor response to neoadjuvant therapy, and NLR > 3.3 correlated with increased Treg infiltration. **Conclusions**: Our data suggest that patients with a high baseline NLR may benefit from intensified neoadjuvant therapy with FOLFIRINOX and radiation. Combination immunotherapy targeting Tregs and the PD1/PDL1 axis may further improve outcomes.

## Simple Summary

Neoadjuvant chemotherapy and chemoradiotherapy have been used in pancreatic adenocarcinoma to downsize tumors for surgical resection and to prevent early metastasis. However, selecting patients who would benefit from a specific regimen remains challenging. In this study, we performed a retrospective analysis of patients treated with FOLFIRINOX or Gemcitabine/nab-paclitaxel (Abraxane) neoadjuvant therapies, with or without radiation, followed by surgical resection, using clinical and pathological data. We observed improved survival with FOLFIRINOX and radiation compared with Gemcitabine/Abraxane among patients with a higher neutrophil-to-lymphocyte ratio (NLR). Poor responders exhibited increased stromal PDL1 and regulatory T cells (Tregs). This study may help patient stratification for neoadjuvant therapy and suggests a potential immunotherapeutic strategy targeting Tregs and the PD1/PDL1 pathway.

## 1. Introduction

Pancreatic ductal adenocarcinoma remains a challenging and aggressive malignancy with poor overall survival, only 13% at 5 years [1]. While 40% of patients present with distant metastases and are not surgical candidates, approximately 15–20% of patients are diagnosed with potentially resectable or borderline resectable disease, while another 30–40% have locally advanced tumors [1]. Tumor location is associated with survival; pancreatic head primaries have slightly improved survival compared to body/tail tumors (median survival 12 vs. 9 months) [2]. Given the poor overall survival and frequent distant disease progression, all patients undergo chemotherapy. Adjuvant chemotherapy is frequently omitted or abbreviated due to operative morbidity or poor functional status. This has led to the increased use of neoadjuvant therapy, even in patients with upfront resectable disease [3]. An additional benefit of neoadjuvant therapy is reducing the rate of post-operative fistula, a major source of surgical morbidity [4]. High-quality evidence to inform optimal perioperative treatment strategies remains limited. Although an increasing number of studies support the potential benefits of neoadjuvant chemotherapy and chemoradiation, interpretation is constrained by significant inter-study heterogeneity, particularly in patient selection criteria and treatment regimens. While a conventional or modified FOLFIRINOX regimen is frequently used in clinical practice, no robust Phase III data has demonstrated the superiority of this approach in the neoadjuvant setting. Retrospective series and meta-analyses suggest that surgery is performed in a quarter to half of patients after FOLFIRINOX, with R0 resection rates exceeding 60% [5,6]. A similar lack of prospective data exists for neoadjuvant Gemcitabine/nab-paclitaxel (Abraxane), with comparisons between FOLFIRINOX and Gemcitabine/Abraxane suggesting equivalent survival, but potentially higher rates of surgery with FOLFIRINOX [7,8,9]. Given the dearth of data for neoadjuvant chemotherapy selection, additional studies are needed to help stratify which patients may benefit from a more aggressive regimen such as FOLFIRINOX.

The tumor microenvironment (TME) of pancreatic adenocarcinoma is characterized by abundant immunosuppressive cells, including regulatory T cells (Tregs), tumor-associated macrophages, and myeloid-derived suppressor cells. The TME also contains a dense fibrotic stroma produced by cancer-associated fibroblasts, which can promote immunosuppression [10]. Depleting immunosuppressive fibroblasts or reprogramming them can render tumors susceptible to cytotoxic or immunotherapy [10,11]. Multiple studies suggest that neoadjuvant therapy has the potential to remodel the TME [12,13,14,15] and that these changes are associated with improved survival outcomes [16].

The addition of immunotherapy to the management of solid tumors has been transformative in oncology. However, the benefit of such therapies has been limited in pancreatic cancer [17,18]. While initially this was attributed to pancreatic cancer being a “cold” tumor type, this assumption has been challenged. We and others have shown that pancreatic cancers have robust T cell infiltrates that are associated with prolonged overall survival [19,20,21]. Further, we found that a subset of pancreatic tumors harbor mature tertiary lymphoid structures associated with high-quality tumor neoantigens and predict for improved overall survival [22]. Several studies are evaluating which immunotherapy combinations may improve neoadjuvant response rates and impart a survival benefit (summarized in [23]). Whether standard-of-care chemotherapy or radiation combinations may help or harm immunotherapeutic interventions, done concurrently or sequentially, remains unknown. We hypothesized that each neoadjuvant treatment regimen would uniquely alter the TME, resulting in opportunities to sequence or combine it with existing immunotherapies.

Several studies have aimed to escalate neoadjuvant therapy with radiation to increase response rates for improved R0 resection and overall survival. These studies have had mixed results. Meta-analyses of several of these trials have demonstrated that the addition of radiation to neoadjuvant chemotherapy is associated with higher R0 resection rates, but does not impact overall survival [24,25]. Therefore, routine use of radiation in the neoadjuvant setting remains controversial but is typically reserved for arterial involvement. It remains unclear which patients may benefit from neoadjuvant chemotherapy or chemoradiotherapy, and understanding how these therapies affect the TME may lead to improved treatment outcomes.

Herein, we report our single-institution data of patients who underwent surgical resection for pancreatic adenocarcinoma following neoadjuvant therapy. To better understand the impact of specific neoadjuvant interventions and identify which patients may benefit from each therapy, we performed several evaluations. Peripheral blood biomarkers prior to treatment, after neoadjuvant therapy, and post-operatively were collected. Resection specimens were analyzed for tumor-infiltrating lymphocytes (TILs) using an AI analysis of scanned whole-slide hematoxylin and eosin (H&E) images. In parallel, we performed multiplex immunohistochemistry and quantified immune cell subsets to validate and compare with the H&E analysis. Finally, we quantified the percent fibrosis on resection specimens. Our data provides preliminary insight into how we might be able to stratify neoadjuvant treatment for patients with non-metastatic pancreatic adenocarcinoma.

## 2. Materials and Methods

### 2.1. Study Population

A retrospective review of all patients who underwent surgical resection for pancreatic ductal adenocarcinoma at Providence Portland Medical Center between 2019 and 2024 was completed under Providence St. Joseph Health IRB Protocol number 2024000994. Inclusion criteria included: age ≥ 18 years, biopsy-proven ductal adenocarcinoma of the pancreas, and having completed at least one cycle of neoadjuvant systemic therapy with or without radiation therapy. Patient demographic and treatment course data was obtained from the electronic medical record. Treatment variables included the type of chemotherapy, number of cycles, dose reductions, and change in treatment type. Laboratory values were obtained from the electronic medical record, including white blood cell count (WBC), absolute neutrophil (ANC) and leukocyte (ALC) counts, hemoglobin, hematocrit, and cancer antigen values. Values for these laboratory data were collected at diagnosis, after neoadjuvant chemotherapy, after neoadjuvant radiation treatment, before surgical resection, and after surgery once surgical drains were removed.

The MR-Linac prospective registry is held under the Providence St. Joseph Health IRB Protocol number 2019000325. Collected information includes diagnosis code, demographic information, and treatment course information, including local control, distant recurrence, survival, and radiation-related toxicity.

### 2.2. Multiplex Immunohistochemistry

Patient surgical specimens were fixed in 10% neutral-buffered formalin, paraffin-embedded and cut at 5 μm for immunohistochemistry (IHC) and multiplex IHC. Six-plex panels of multiplex IHC were performed as follows. Primary antibodies diluted in blocking/diluent buffer (Akoya Biosciences, Marlborough, MA, USA) were sequentially applied for 15 min to 60 min at room temperature, depending on the antibody, at the following concentrations: anti-CD8 (1:400, Abcam, SP16, Waltham, MA, USA), anti-CD163 (1:4, Ventana, 760-4437, Tucson, AZ, USA), anti-cytokeratin (CK) (1:3000, Dako, M3515, Carpinteria, CA, USA), anti-CD3 (1:600, GeneTex, GTX16669, Irvine, CA, USA), anti-FoxP3 (1:400, Abcam, ab20034), anti-PDL1(1:1600, Cell Signaling, 13684, Danvers, MA, USA). MACH-2 anti-rabbit or anti-mouse HRP-conjugated polymer (Biocare Medical, Pacheco, CA, USA) was incubated for 10 min at room temperature. Fluorescent signal was amplified by incubation with tyramide signal amplification (TSA)-conjugated Opal dyes (Akoya Biosciences) for 10 min (60 min for Opal780) at room temperature using Opal-520, Opal-570, Opal-620, Opal-690, Opal780 and Aluora 430 (Thermo Fisher Scientific, Waltham, MA, USA). Nuclei were counterstained with DAPI. Whole tissues were scanned at 20× using an Akoya Fusion microscope, and at least 5 regions of interest per tissue section were analyzed by inForm v3.0.0 (Akoya Biosciences). The six-plex panels were analyzed using tissue segmentation (tumor and stroma), cell segmentation, and cell phenotyping. Quantified cell populations comprised CD8^+^ T cells, CD4^+^ T cells (CD3^+^CD8^−^FoxP3^−^ cells), regulatory T cells (Tregs) (CD3^+^FoxP3^+^), macrophages (CD163^+^ cells), PDL1 single^+^ cells, CD163^+^PDL1^+^ cells, and CK^+^ PDL1^+^ cells.

### 2.3. Masson’s Trichrome Staining and Analysis

After deparaffinization, Masson’s Trichrome staining was performed following the manufacturer’s protocol (Newcomer Supply). Slides were scanned at 20× using a Hamamatsu Nanozoomer scanner(Hamamatsu Photonics, Hamamatsu, Japan). Whole tissue images were quantified by QuPathv 0.6.0 [26]. Briefly, the tissue area was manually annotated, and thresholds were applied within the annotated area to identify tissue-positive regions. The percentage of collagen-positive area stained blue was measured by creating threshold and classification.

### 2.4. H&E TIL Analysis

After deparaffinization, hematoxylin and eosin (H&E) staining and coverslipping were performed using Tissue-Tek Prisma and Tissue-Tek Film (Sakura, Torrance, CA, USA), respectively. Slides were scanned at 40× with a Hamamatsu Nanozoomer scanner, and whole-tissue images were analyzed in QuPath. Tissue annotation was performed as described for Masson’s Trichrome staining analysis, and TIL-positive tiles were classified using the pancancer-lymphocytes-inceptionv4.tcga model in WSInfer v 0.4.0 Extension [27].

### 2.5. Statistics

Statistical comparisons were performed using GraphPad Prism v 10.5. Comparisons between the proportion of patients with a specified outcome were performed using Fisher’s exact test. Kaplan–Meier survival analyses were performed using Wilcoxon comparisons between groups. Wilcoxon test was selected over log-rank due to the emphasis on early events, which is more relevant in this population where many survival events occur at early time points. Differences in laboratory values or cell counts between groups were compared using one-way ANOVA with Tukey’s correction, two-way ANOVA with Sidak’s correction, or, for non-parametric measures, Kruskal–Wallis test with Dunn’s correction for multiple comparisons. When comparing two groups, we utilized unpaired *t*-tests. Statistical significance is noted in each figure and legend.

## 3. Results

Between 2019 and 2024, 104 patients underwent resection for pancreatic ductal adenocarcinoma. Of those, 79 patients received neoadjuvant therapy for inclusion in this study (Table 1). There were slightly more male patients (54%) with racial demographics mirroring those of our catchment area. Eighteen percent of patients were considered resectable at baseline, while 67% were borderline resectable, and 5% were considered locally advanced/unresectable. All 79 patients received neoadjuvant chemotherapy: 61 patients (77%) with FOLFIRINOX and 18 patients (23%) with Gemcitabine/Abraxane. Two of the patients who started FOLFIRINOX required a change of treatment to Gemcitabine/Abraxane, and were included in the FOLFIRINOX cohort for this analysis. Multidisciplinary tumor board discussion regarding concern for resectability, particularly arterial involvement, after neoadjuvant chemotherapy resulted in patients undergoing radiation therapy (RT). A subset of 14 patients (18%) also underwent neoadjuvant radiation therapy. Eleven of the 14 (79%) with MR-guided stereotactic ablative radiotherapy (mrSBRT) and three (21%) completed standard fractionated chemoradiation to 50 Gy in 25–28 fractions. While not included in this study population, we have a prospective registry for patients undergoing mrSBRT, and during the study period, 48 pancreatic cancer patients were radiated, including those considered unresectable, suggesting approximately one fourth of patients treated with mrSBRT ultimately underwent resection.

Surgical outcomes are summarized in Table 2. The majority of patients underwent pancreaticoduodenectomy (70%). Seventy-five percent of patients had negative surgical margins. Adverse pathologic features, such as lymphovascular space invasion (LVSI), were present in 33% of patients, while 61% of tumors had perineural invasion (PNI). Tumor regression (inclusive of pathologic complete response (pCR), near-complete response [TRG1], or partial tumor regression [TRG2]) was seen in 59% of patients, with 4% having a pCR.

**Figure 1:**
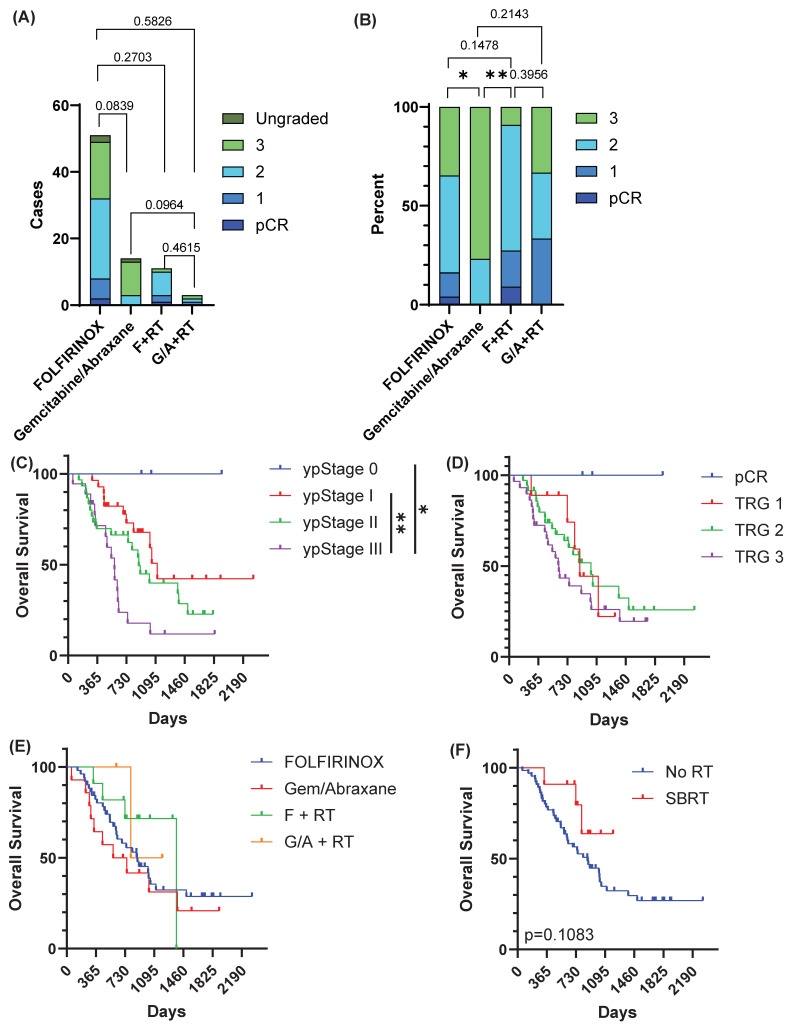
Superior treatment response with FOLFIRINOX and radiation. (**A**) Tumor regression grade (TRG) of each tumor based on which neoadjuvant therapy they received. F + RT = FOLFIRINOX followed by radiation (RT). G/A + RT = Gemcitabine/Abraxane followed by RT. pCR = pathologic complete response. (**B**) TRG percentage of total, excluding tumors that were ungraded (*n* = 3). Statistical comparisons were performed between proportions of responders (pCR, TRG1, TRG2) and non-responders (TRG3). (**C**) Overall survival based on pathologic TNM stage grouping. yp = TNM stage based on surgical pathology (p) in a patient who received neoadjuvant therapy (y). (**D**) Overall survival stratified by TRG. (**E**) Overall survival based on neoadjuvant therapy received. (**F**) Overall survival of patients who received stereotactic body radiotherapy (SBRT) compared to patients who did not receive radiation (No RT). * *p* < 0.05, ** *p* < 0.01.

To evaluate whether pre-operative therapy impacted the degree of tumor regression, we compared tumor regression grade (TRG) between treatment cohorts (Figure 1). While the absolute numbers (Figure 1A) and proportions (Figure 1B) of tumors with each TRG were not statistically different across cohorts, when evaluated as a binary (regression [pCR + TRG1 + TRG2] vs. no regression [TRG 3]), there were significant differences between groups (statistics displayed in Figure 1B). Tumor regression was more frequent in patients who received FOLFIRINOX compared to patients who received Gemcitabine/Abraxane (65% vs. 23%, *p* = 0.0105). The addition of radiation increased rates of tumor regression, with 91% of patients who received FOLFIRINOX followed by RT (F + RT) having tumor regression, and 67% of patients who received Gemcitabine/Abraxane followed by RT having tumor regression, but there was no difference between radiation cohorts (*p* = 0.3956).

We then evaluated overall survival based on the neoadjuvant treatment regimen and response. Overall survival was positively associated with overall TNM stage grouping based on pathological staging post-neoadjuvant therapy (yp) (Figure 1C). Median survival was not reached for patients with pCR (ypStage 0, *n* = 3) (Figure 1C). Patients with ypStage I (*n* = 28) had improved survival over patients with ypStage III (*n* = 18) (median survival 1115 days vs. 580 days, *p* = 0.0012), though no statistical difference was observed between those with ypStage II (*n* = 30) (median survival 884 days, *p* = 0.1014 vs. ypStage I; *p* = 0.1266 vs. ypStage III) (Figure 1C). We next evaluated whether TRG could stratify patient survival (Figure 1D). While pCR showed excellent survival, TRG1-TRG3 failed to separate survival with any statistical significance (Figure 1D). The neoadjuvant treatment regimen did not statistically impact survival; however, there were trends showing improved survival for FOLFIRINOX over Gemcitabine/Abraxane (HR 0.76 [95% CI 0.35–1.65], *p* = 0.3125, Figure 1E), and for radiation regimens over chemotherapy alone (F vs. F + RT: HR 1.77 [95% CI 0.75–4.16], *p* = 0.2271, G/A vs. G/A + RT: HR 2.60 [95% CI 0.60–11.19], *p* = 0.2359, Figure 1E). To further evaluate the impact of radiation on survival, we compared patients who received mrSBRT against patients who received chemotherapy alone (Figure 1F). Patients who underwent mrSBRT had a trend towards improved survival (median survival not reached vs. 873 days, *p* = 0.1083, Figure 1F).

**Figure 2:**
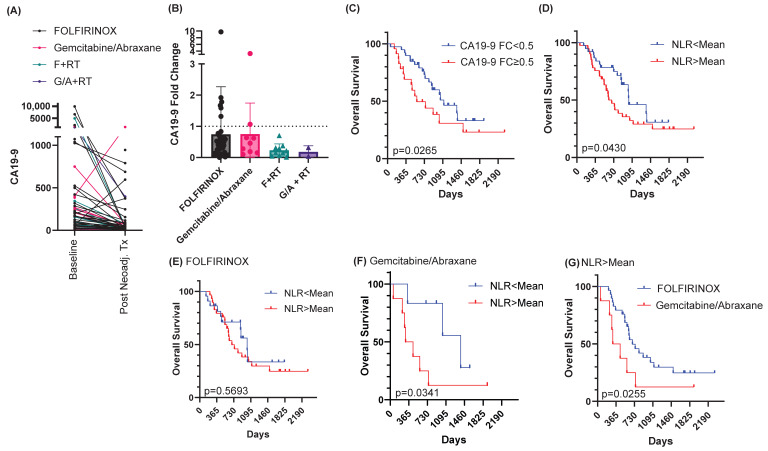
Peripheral blood biomarkers correlate with overall survival. (**A**) Individual patient changes in CA19-9 with neoadjuvant treatment. F + RT = FOLFIRINOX followed by RT. G/A + RT = Gemcitabine/Abraxane followed by RT. (**B**) Fold change in CA19-9 between baseline and post-neoadjuvant treatment, where <1.0 (dotted line) means a reduction in CA19-9 with therapy. Data represent the mean ± standard deviation. (**C**) Overall survival stratified by CA19-9 fold change (FC) less than 0.5. (**D**) Overall survival stratified by mean neutrophil-to-lymphocyte ratio (NLR). (**E**) Overall survival in patients receiving FOLFIRINOX based on NLR. (**F**) Overall survival in patients receiving Gemcitabine/Abraxane based on NLR. (**G**) Overall survival of patients with an NLR > mean, stratified by neoadjuvant chemotherapy regimen.

We next evaluated if there were any peripheral blood markers that changed with treatment and were predictive of outcomes. As expected, there was a trend towards reduction in the tumor marker CA19-9 after neoadjuvant treatment, regardless of treatment cohort (Figure 2A,B). Radiation cohorts had greater reductions in CA19-9, but this was not statistically different from non-radiation cohorts (Figure 2A,B). Using a cutoff of a two-fold reduction in CA19-9 (FC = 0.5), overall survival was greater in patients with a greater reduction in CA19-9 (Figure 2C, *p* = 0.0265). The neutrophil-to-lymphocyte ratio (NLR) has been extensively studied as a marker of prognosis for patients with pancreatic ductal adenocarcinoma [28]. There were no differences in the baseline NLR by group, nor was the NLR significantly altered by neoadjuvant regimen (Supplementary Figure S1A). However, an NLR less than the mean (mean NLR = 3.3) was associated with improved overall survival (median survival 1028 days vs. 633 days, *p* = 0.0430, Figure 2D). We then evaluated overall survival stratified by neoadjuvant regimen selection and baseline NLR values (Figure 2E,F). Baseline NLR was not associated with survival in patients treated with FOLFIRINOX (median survival 1017 days vs. 691 days, *p* = 0.5693, Figure 2E), but did stratify survival in patients treated with Gemcitabine/Abraxane (median survival 1385 days vs. 373.5 days, *p* = 0.0341, Figure 2F). Visualizing this differently, for patients with a baseline NLR greater than the mean, survival was improved by neoadjuvant FOLFIRINOX compared to Gemcitabine/Abraxane (median survival 691 days vs. 373.5 days, *p* = 0.0255, Figure 2G). Patients who had a baseline NLR less than the mean had much improved survival regardless of the neoadjuvant chemotherapy regimen (median survival 1017 days vs. 1385 days, *p* = 0.4274, Supplementary Figure S1B). Though our sample size is small for patients who underwent radiation, overall survival trended towards improvement in those individuals with an NLR greater than the mean who received radiation compared to those who had chemotherapy alone (median survival not reached; Supplementary Figure S1C,D). Consistent with prior publications, our data suggests patients with a higher baseline NLR have worse outcomes. Uniquely, our data suggests that escalating neoadjuvant therapy with either FOLFIRINOX over Gemcitabine/Abraxane, or the addition of radiation, may improve survival in this population of patients.

**Figure 3:**
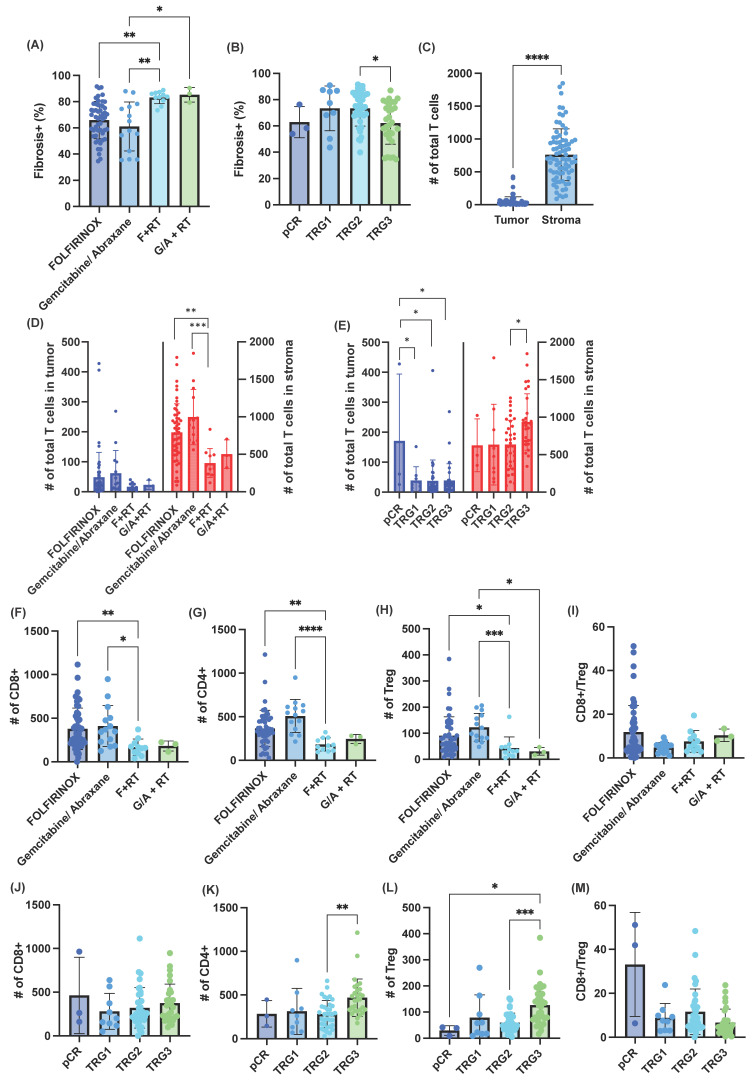
Tumor microenvironment alterations with neoadjuvant treatment. (**A**) Percentage of fibrosis based on neoadjuvant treatment regimen. (**B**) Percentage of fibrosis based on tumor regression grade (TRG). pCR = pathologic complete response. Quantification of total T cells, including CD8^+^ T cells, CD4^+^ T cells (CD3^+^CD8^−^FoxP3^−^), and regulatory T cells (Tregs) (CD3^+^FoxP3^+^), using multiplex immunohistochemistry (**C**), stratified by neoadjuvant treatment (**D**) or TRG (**E**). Quantification of tumor-infiltrating (**F**) CD8^+^ T cells, (**G**) CD4^+^ T cells, and (**H**) Tregs based on neoadjuvant treatment. (**I**) Tumor-infiltrating CD8-to-Treg ratio based on neoadjuvant treatment. Quantification of (**J**) CD8^+^ T cells, (**K**) CD4^+^ T cells, and (**L**) Tregs based on TRG. (**M**) Tumor-infiltrating CD8-to-Treg ratio based on TRG. Data represent the mean ± standard deviation. * *p* < 0.05, ** *p* < 0.01, *** *p* < 0.001, **** *p* < 0.0001.

Next, we focused on immunohistochemical biomarkers from the resection specimens. A common feature of pancreatic adenocarcinoma is a fibrotic stroma. This can represent unfavorable baseline biology, or it may represent a response to neoadjuvant therapy. We quantified the percentage of fibrosis using trichrome staining (Figure 3A, Supplementary Figure S2A). Patients who received neoadjuvant radiation had significantly more fibrosis than patients who received chemotherapy alone (Figure 3A), consistent with the known fibrotic effects of radiation [29]. TRG3 had the least fibrosis, suggesting tumor regression may also contribute to fibrosis (Figure 3B). We next utilized two methods for quantifying tumor-infiltrating lymphocytes (TILs). First, we used H&E slides and quantified TIL for the whole slide using the WSInfer plug-in for QuPath (Supplementary Figure S2B). Neoadjuvant radiation decreased TILs at the time of surgical resection (Supplementary Figure S3A,B). No differences in TILs were observed between different TRGs (Supplementary Figure S3C). We also performed multiplex IHC on a serial slide to the H&E slide, and stained for CD3, CD8, FoxP3, CD163, cytokeratin (CK), PDL1, and DAPI nuclear stain (Supplementary Figure S2(Ci)). Quantification was performed on at least five regions of interest per slide. InForm analysis allowed for discrimination of “tumor” (includes normal ducts and tumor, identified as all CK^+^ regions) versus stroma (Supplementary Figure S2(Cii)), and demonstrated that the vast majority of TILs were in the stroma (Figure 3C, Supplementary Figure S2(Civ)). Consistent with the TIL analysis using H&E, fewer TILs were observed in patients who underwent neoadjuvant radiation (Figure 3D, Supplementary Figure S3D). Interestingly, when evaluating by tissue compartment, specimens with pCR had the most epithelial TILs, where TRG3 had the most stromal TILs (Figure 3E). Using more specific cell phenotyping, radiation was associated with reductions in CD8^+^, CD4^+^ T cells, and Tregs (Figure 3F–H). However, CD8:Treg ratios remained similar between groups (Figure 3I). When evaluating T cell subtypes by TRG, there were no differences in CD8^+^ T cells (Figure 3J), but there were more CD4^+^ T cells with decreasing response to treatment, with TRG3 having the highest levels of CD4^+^ infiltrates (Figure 3K). Consistent with this, there were higher levels of inhibitory Tregs in patients with worse treatment responses (Figure 3L). While not statistically significant, CD8:Treg ratios trended toward being highest in pCR and lowest in TRG3 (pCR vs. TRG3, *p* = 0.0782; TRG2 vs. TRG3, *p* = 0.0527, Figure 3M).

**Figure 4:**
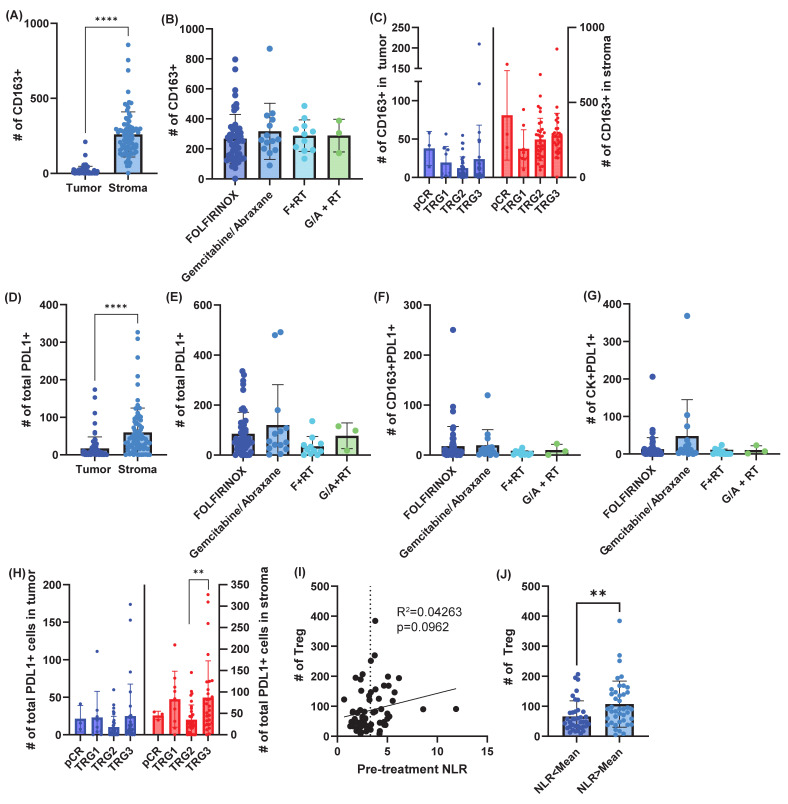
Immune regulatory molecules associate with treatment response. (**A**–**C**) Quantification of tumor-associated macrophages (CD163^+^) by tissue compartment (**A**), by neoadjuvant therapy received (**B**), and by tumor regression grade (TRG) (**C**). pCR = pathologic complete response. (**D**) PDL1^+^ cells based on tumor or stromal compartment. (**E**) PDL1 expression based on neoadjuvant treatment regimen. (**F**) Quantification of PDL1^+^ tumor-associated macrophages (CD163^+^PDL1^+^) by neoadjuvant regimen. (**G**) Quantification of PDL1^+^ epithelial cells (CK^+^PDL1^+^) by treatment regimen. (**H**) PDL1 expression based on TRG. (**I**) Correlation between baseline neutrophil-to-lymphocyte ratio (NLR) and tumor-infiltrating regulatory T cells (Tregs) (CD3^+^FoxP3^+^). Dashed line at 3.3 represents the mean NLR and cutoff utilized for this study. (**J**) Quantification of Tregs based on baseline NLR. Data represent the mean ± standard deviation. ** *p* < 0.01, **** *p* < 0.0001.

Tumor-associated macrophages were enriched in the stromal compartment, as expected (Figure 4A). There was no difference in macrophages by neoadjuvant treatment regimen (Figure 4B) or TRG (Figure 4C). We next evaluated the immune checkpoint ligand, PDL1 expression. PDL1 was more frequently expressed in the stromal compartment (Figure 4D), but was not significantly different between neoadjuvant treatment regimens (Figure 4E–G). PDL1 expression was higher in the stroma of patients whose tumors failed to respond to therapy (TRG3, Figure 4H). Taken together, the immune infiltrate data indicate that pancreatic cancer has an active immune response, with tumor treatment response inversely correlating with levels of Tregs and stromal PDL1^+^ cells, suggesting immune-mediated clearance of tumor cells may have been impaired in patients with poorly responsive tumors. To determine if there was a peripheral blood marker that might indicate which tumors were predisposed to poor immune-mediated tumor clearance, we evaluated correlations between peripheral blood markers and immune cell counts. No peripheral blood markers correlated with tumor-infiltrating immune cell counts. However, there was a trend towards a correlation between baseline NLR and infiltrating Tregs (*p* = 0.0962, Figure 4I). Using the mean value as a cutoff, there were more Tregs infiltrating the tumors of patients with an NLR > 3.3 (Figure 4J). These data support the NLR as a peripheral biomarker of poorly responsive tumors.

## 4. Discussion

Improving outcomes for patients with pancreatic adenocarcinoma remains a critical unmet need. In particular, optimizing neoadjuvant treatment strategies to maximize tumor regression and increase the likelihood of successful surgical resection could meaningfully improve survival and cure rates among patients with resectable locoregional disease. In this analysis, we evaluated the response to neoadjuvant therapy at our single institution from 2019 to 2024 using modern treatment regimens. Our study is retrospective in nature and should be cautiously interpreted. In addition to an improved response rate, the FOLFIRINOX regimen was associated with a trend, albeit nonsignificant, towards longer survival, which may be subject to patient selection bias as Gemcitabine/nab-paclitaxel are commonly administered to patients with poorer performance status. The addition of mrSBRT to ablative doses (50 Gy in 5 fractions, BED_10_ = 100) improved response and survival, but the small number of patients included in the analysis limited statistical significance. The addition of radiation to neoadjuvant chemotherapy has been extensively studied with mixed results. The LAP07 study compared chemotherapy with Gemcitabine alone versus Gemcitabine followed by chemoradiation (54 Gy in 30 fractions with capecitabine, BED_10_ = 63.72) in patients with locally advanced pancreatic cancer, and demonstrated no difference in overall survival; however, there was a trend toward improved progression-free survival in the chemoradiation arm (8.4 vs. 9.9 months, *p* = 0.06) with reduced locoregional recurrence (46% vs. 32%, *p* = 0.04) [30]. The PREOPANC trial compared neoadjuvant Gemcitabine followed by chemoradiation (36 Gy in 15 fractions with Gemcitabine, BED_10_ = 44.64) and surgery versus upfront surgery followed by adjuvant Gemcitabine in resectable or borderline resectable pancreatic cancer [31]. Neoadjuvant chemoradiation improved R0 resection rates, and the long-term follow-up demonstrated an overall survival benefit (15.7 vs. 14.3 months, *p* = 0.025) [32]. In the Alliance A021501 trial of borderline resectable pancreatic cancer, the addition of moderate-dose SBRT (25–33 Gy in 5 fractions, BED_10_ = 37.5–54.78) to FOLFIRINOX did not improve outcomes [33]. However, the SMART trial utilizing stereotactic MR-guided on-table adaptive radiation therapy (50 Gy in 5 fractions, BED_10_ = 100) for borderline resectable (57%) and locally advanced (43%) pancreatic cancer after three months of chemotherapy (82% FOLFIRINOX, 17% Gemcitabine/Abraxane) achieved a 35% operative rate, 2-year local control rate of 78.2% and 2-year overall survival (OS) of 53.6% [34]. Together, these data suggest that differences in radiation technique that allow for truly ablative doses (BED_10_ ≥ 100) may result in superior local control, which can in turn impact progression-free survival. Additionally, patient selection, such as borderline versus locally advanced pancreatic cancer, may also affect the benefit of radiotherapy, as locally advanced pancreatic cancer may require additional therapy for control, while chemotherapy alone may be sufficient for borderline resectable disease. Finally, chemotherapy choice differed among these trials, suggesting that the addition of radiation to Gemcitabine-based chemotherapy was more advantageous, which is consistent with our retrospective cohort observations. Our data add to these studies, and provide additional support for a role for radiation in select patients with pancreatic adenocarcinoma. Further investigations are needed to understand when to alter the current treatment strategy and select resectable or borderline resectable patients for radiation in clinical practice.

Tumor bed fibrosis was increased in patients who received radiation. Fibrosis is a well-characterized late effect of radiation [29] and is often a deleterious side effect. However, pancreatic fibrosis is associated with reduced rates of pancreatic fistulas [35,36], a serious complication of tumor resection. Multivariate analyses demonstrated an association between neoadjuvant chemotherapy and neoadjuvant radiation with reduced rates of pancreatic fistulas [4]. It remains unclear whether fibrosis resulting from tumor regression or fibrosis induced by treatment modality (e.g., radiation) impacts fistula risk differently. Nevertheless, these data may provide further support for neoadjuvant radiation, which has been associated with increased fibrosis, thereby potentially reducing the risk of a post-operative fistula.

We evaluated the well-characterized biomarker NLR for predicting treatment response. Numerous groups have previously published the prognostic utility of the NLR in pancreatic cancer, with a meta-analysis of 1804 patients indicating that a high NLR was associated with reduced overall survival, reduced cancer-specific survival, poor tumor differentiation, high CA19-9, and poor performance status [28]. One consistent challenge with the NLR is determining the cut-point for “high” versus “low”, which varied across studies from 2.3 to 5 [28]. We selected a cut-point of 3.3, which was the mean NLR in our cohort, which compares favorably with the studies included in the meta-analysis. In our cohort of patients with a high NLR, FOLFIRINOX or the addition of radiation was associated with improved survival. In contrast, in patients with a low NLR, outcomes were similar regardless of the neoadjuvant regimen. These data may provide additional selection criteria for who should be directed toward mrSBRT prior to resection.

Finally, our data provide additional evidence that pancreatic cancer is not immune- quiescent. Our group has previously demonstrated that a subset of pancreatic tumors develops spontaneous tertiary lymphoid structures, with prolonged overall and progression-free survival associated with high-quality tumor neoantigens and increased humoral immunity [22]. Our current analysis demonstrates that tumors that failed to respond to neoadjuvant therapy had higher levels of suppressive Tregs and stromal PDL1 expression. Without pretreatment tissue, we are unable to determine whether these tumors failed to respond because they harbored these unfavorable features at baseline, or whether these features were enriched by treatment and lack of response/tumor progression. Extrapolating from our prior publications with upfront-resected patients who did not undergo neoadjuvant therapy [21,22,37], our cohort had higher levels of CD3^+^ and CD8^+^ T cell infiltrates and a higher percentage of PDL1^+^ cells, suggesting neoadjuvant therapy modulated these TME features. Consistent with the other unfavorable features that correlate with a higher NLR, we found that Treg infiltrates are also associated with elevated baseline NLR. Targeting Tregs using αCTLA-4 antibodies, such as ipilimumab, has failed as a monotherapy approach or in combination with αPD1/αPDL1 in pancreatic cancer [18,38]. Combining αCTLA-4 and αPDL1 with concurrent Gemcitabine/Abraxane also failed to improve overall survival in metastatic pancreatic cancer patients [39]. Preclinical models have shown improved response rates with Treg-targeting agents in combinations. Combination αIL-6 and αCTLA-4 improved immune cell infiltration and function, resulting in reduced pancreatic tumor size [40]. αCD40 agonist antibodies have been used with chemotherapy prior to dual checkpoint blockade with αCTLA-4 and αPD1 [41]. Several ongoing clinical trials are evaluating combination immunotherapy with cytotoxic therapy (recently reviewed in [23]). Our data suggest that within non-metastatic pancreatic cancer, select patients may benefit from sequential therapy, such as αCTLA-4 and αPD1 after neoadjuvant chemotherapy and mrSBRT, if the baseline NLR is unfavorable and serial imaging suggests poor treatment response. Improving outcomes for patients with pancreatic cancer remains a critical need, and tailoring our treatment approach based on clinicopathologic features can be an effective starting point.

## 5. Conclusions

Our study suggests that a baseline NLR can be used not only to predict prognosis, but also to select patients for specific neoadjuvant therapies for non-metastatic pancreatic adenocarcinoma. An elevated NLR was associated with increased Treg infiltration and poor response to neoadjuvant therapy. Neoadjuvant therapy with FOLFIRINOX and/or the addition of radiation may be beneficial for patients with a high baseline NLR. Further studies are warranted to evaluate immunotherapeutic strategies targeting Tregs and the PD1/PDL1 pathway in conjunction with cytotoxic neoadjuvant regimens.

## Figures and Tables

**Table 1:** Patient characteristics.

	***n*** **= 79**
**Median** **age, years**	67 [Range: 44–84]
**Sex, number (%)**	
Male	43 (54%)
Female	36 (46%)
**Race**	
White	68 (86%)
Asian	1 (1%)
Black	2 (3%)
Other	8 (10%)
**Resectability**	
Resectable	14 (18%)
Borderline resectable	53 (67%)
Locally advanced	4 (5%)
Unclassified	8 (10%)
**Median tumor marker CA19-9**	
At diagnosis	139.5 [Range: 3–9940]
Pre-surgery	32 [Range: 2–1315]
**Neoadjuvant chemotherapy**	
FOLFIRINOX	61 (77%)
Gemcitabine/Abraxane	18 (23%)
**Dose reduction required**	47 (59%)
**Radiation**	14 (18%)
mrSBRT 50Gy/5 fractions	11 (79%)
chemoRT 50Gy/25–28 fractions	3 (21%)

**Table 2:** Surgical outcomes.

Variable	
**Histopathological Finding**	***n*** **(%)**
**Surgery** Performed *	
Pancreaticoduodenectomy	55 (70%)
Subtotal pancreatoduodenectomy	7 (9%)
Distal pancreatectomy with splenectomy	8 (10%)
Subtotal distal pancreatectomy with splenectomy	7 (9%)
Not specified	2 (3%)
**Surgical Margin Status**	
Negative	59 (75%)
Positive	20 (25%)
Pathologic T Stage *	
ypT0	3 (4%)
ypT1	33 (42%)
ypT2	33 (42%)
ypT3	8 (10%)
ypT4	2 (3%)
Pathologic N Stage *	
ypN0	36 (46%)
ypN1	26 (33%)
ypN2	17 (22%)
**Number of nodes removed, median**	19 [Range: 1–31]
Pathologic Stage Grouping *	
0	3 (4%)
IA	14 (18%)
IB	14 (18%)
IIA	4 (5%)
IIB	26 (33%)
III	18 (23%)
**Adverse pathologic features**	
LVSI	26 (33%)
PNI	48 (61%)
**Grade**	
Grade 1	2 (3%)
Grade 2	41 (52%)
Grade 3	27 (34%)
Not reported	9 (11%)
**Tumor Regression Grade**	
pCR (complete response)	3 (4%)
1 (near complete response, single cells or rare small groups of cancer cells)	9 (11%)
2 (Partial tumor regression but more than single cells or rare small groups of cancer cells)	35 (44%)
3 (No or poor response)	29 (37%)
**Not reported**	3 (4%)

* Percentages may not total 100 due to rounding.

## Data Availability

The data are not publicly available due to privacy or ethical restrictions. The deidentified data are available from the corresponding author upon reasonable request.

## Supplementary Materials

The following supporting information can be downloaded at: https://www.mdpi.com/article/10.3390/cancers18101567/s1, Figure S1: Factors associated with improved treatment response and survival. (A) Neutrophil-to-lymphocyte ratio (NLR) changes by neoadjuvant treatment regimen. F + RT = FOLFIRINOX followed by RT. G/A + RT = Gemcitabine/Abraxane followed by RT. Data represent the mean ± standard deviation. (B) Overall survival in patients with NLR < Mean based on neoadjuvant chemotherapy regimen. (C,D) Overall survival in patients with NLR > Mean who received FOLFIRINOX +/− RT (C) or Gemcitabine/Abraxane +/− RT (D); Figure S2: The process of immunohistochemical analysis of resected specimen. (A) Masson’s trichrome analysis. (i) Tissue surrounding area was annotated manually with a yellow line to exclude artifact. (ii) Collagen-positive areas stained blue were measured by applying thresholding and classification within tissue positive regions delineated by the yellow line. Cyan = Collagen-positive, Purple = Collagen-negative. (B) H&E TIL analysis. (i) Tissue surrounding area was annotated manually with a yellow line to exclude artifacts. (ii) Tissue-positive regions were processed using the WSInfer Extension and segmented into tiles. (iii) Tiles were classified as either lymphocytes or non-lymphocytes. Each tile is approximately 50 μm × 50 μm. (C) Multiplex IHC analysis. (i) A region of interest from multiplex IHC is shown. CD8 = yellow, CD3 = magenta, FoxP3 = pink, CD163 = cyan, PDL1 = red, cytokeratin (CK) = green, DAPI = blue. (ii–iv) Multiplex images were analyzed using inForm software. Images were quantified through tissue segmentation (ii), cell segmentation (iii), and phenotyping (iv). (ii) CK^+^ regions were classified as “tumor” in green. (iii) Cells were segmented using DAPI. (iv) Cell types were phenotyped as follows: CD8^+^ = yellow, CD3^+^ = magenta, FoxP3^+^ = pink, CD163^+^ = cyan, PDL1 single^+^ = red, CD163^+^ PDL1^+^ = orange, CK^+^PDL1^+^ = brown, CK^+^ = green, others = blue; Figure S3: Tumor infiltrating lymphocyte quantification. (A) Percent of cells that are lymphocytes based on H&E analysis. (B,C) Number of lymphocyte-positive tiles by neoadjuvant regimen (B) and by tumor regression grade (TRG) (C) using H&E analysis. pCR = pathologic complete response. (D) Number of total T cells by neoadjuvant regimen based on multiplex immunohistochemistry analysis. RT = radiation. * *p* < 0.05, ** *p* < 0.01, **** *p* < 0.0001.
