# Supplementary material for: The Differential Impact of Neoadjuvant Therapies on the Tumor Microenvironment, Peripheral Biomarkers, and Survival in Pancreatic Cancer: A Retrospective Cohort Study
Source: Cancers (Basel). 2026 May 12;18(10):1567. doi: 10.3390/cancers18101567 (PMC13204183; doi:10.3390/cancers18101567)
Supplement: Supplementary file 1 [file cancers-18-01567-s001.zip › cancers-4265321-supplementary.pdf]

Supplementary Figure S1.

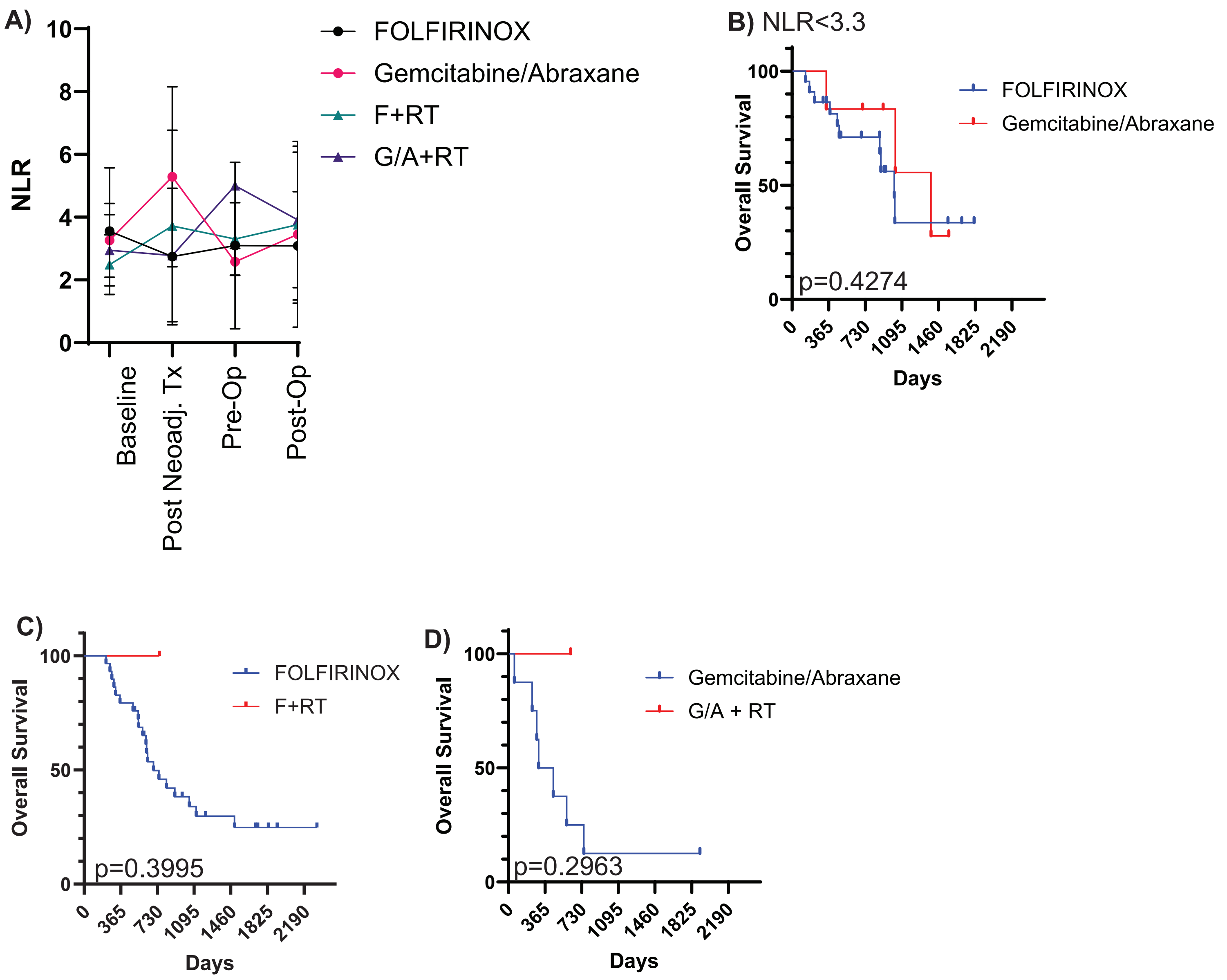

Supplementary Figure S1. Factors associated with improved treatment response and survival. A) Neutrophil-to-lymphocyte ratio (NLR) changes by neoadjuvant treatment regimen. F + RT = FOLFIRINOX followed by RT. G/A + RT = Gemcitabine/Abraxane followed by RT. Data represent the mean ± standard deviation. B) Overall survival in patients with NLR < Mean based on neoadjuvant chemotherapy regimen. C,D) Overall survival in patients with NLR > Mean who received FOLFIRINOX +/− RT C) or Gemcitabine/ Abraxane +/− RT D).

Supplementary Figure S2

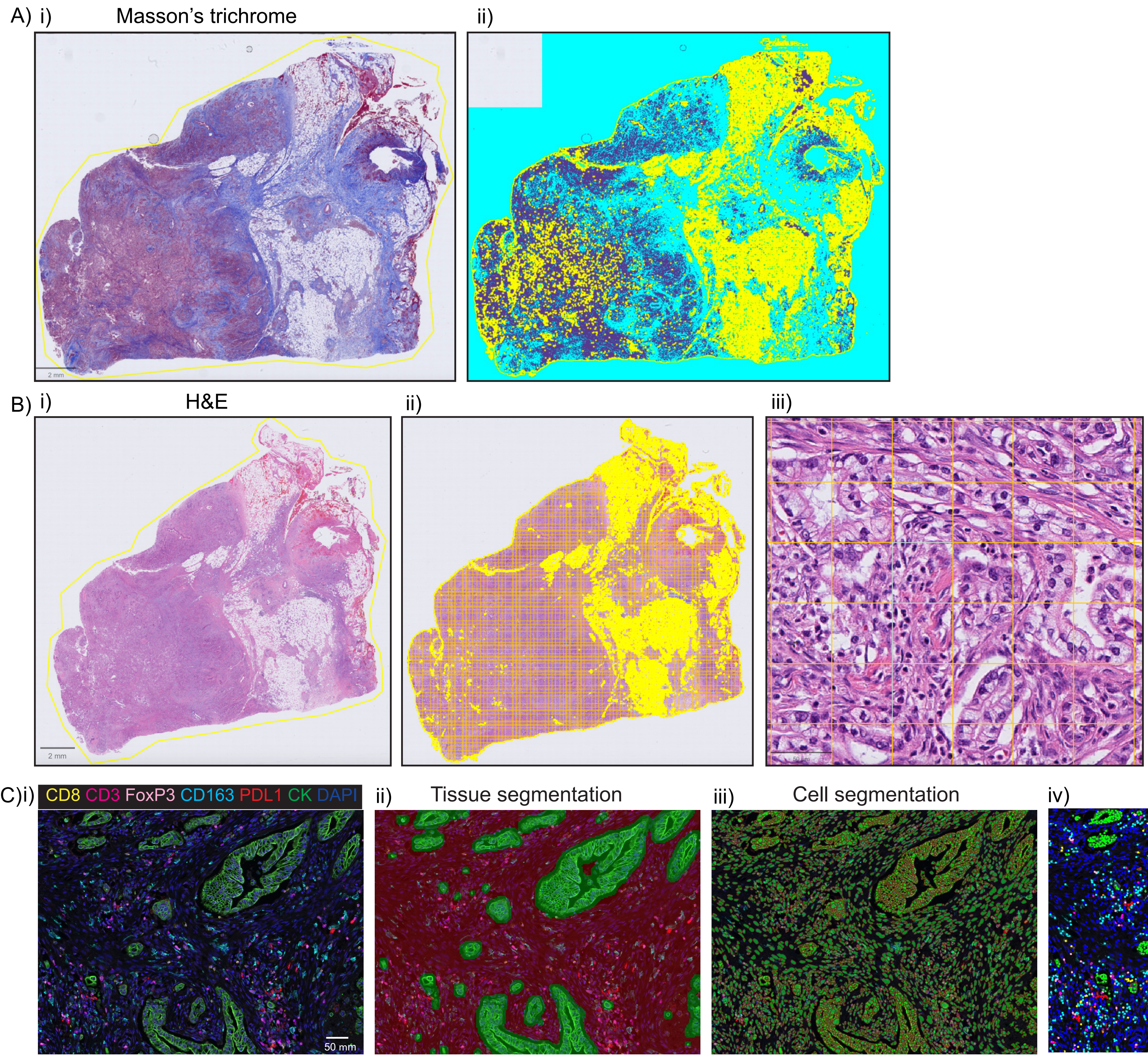

Supplementary Figure S2. The process of immunohistochemical analysis of resected specimen. A) Masson's trichrome analysis. i) Tissue surrounding area was annotated manually with a yellow line to exclude artifact. ii) Collagen-positive areas stained blue were measured by applying thresholding and classification within tissue positive regions delineated by the yellow line. Cyan= Collagen-positive, Purple= Collagen-negative. B) H&E TIL analysis. i) Tissue surrounding area was annotated manually with a yellow line to exclude artifacts. ii) Tissue-positive regions were processed using the WSInfer Extension and segmented into tiles. iii) Tiles were classified as either lymphocytes or non-lymphocytes. Each tile is approximately 50 µm × 50 µm. C) Multiplex IHC analysis. i) A region of interest from multiplex IHC is shown. CD8= yellow, CD3= magenta, FoxP3= pink, CD163= cyan, PDL1= red, cytokeratin (CK)= green, DAPI= blue. ii-iv) Multiplex images were analyzed using inForm software. Images were quantified through tissue segmentation (ii), cell segmentation (iii), and phenotyping (iv). ii) CK+ regions were classified as "tumor" in green. iii) Cells were segmented using DAPI. iv) Cell types were phenotyped as follows: CD8+ = yellow, CD3+ = magenta, FoxP3+ = pink, CD163+ = cyan, PDL1 single+ = red, CD163+ PDL1+ = orange, CK+PDL1+ = brown, CK+ = green, others= blue.

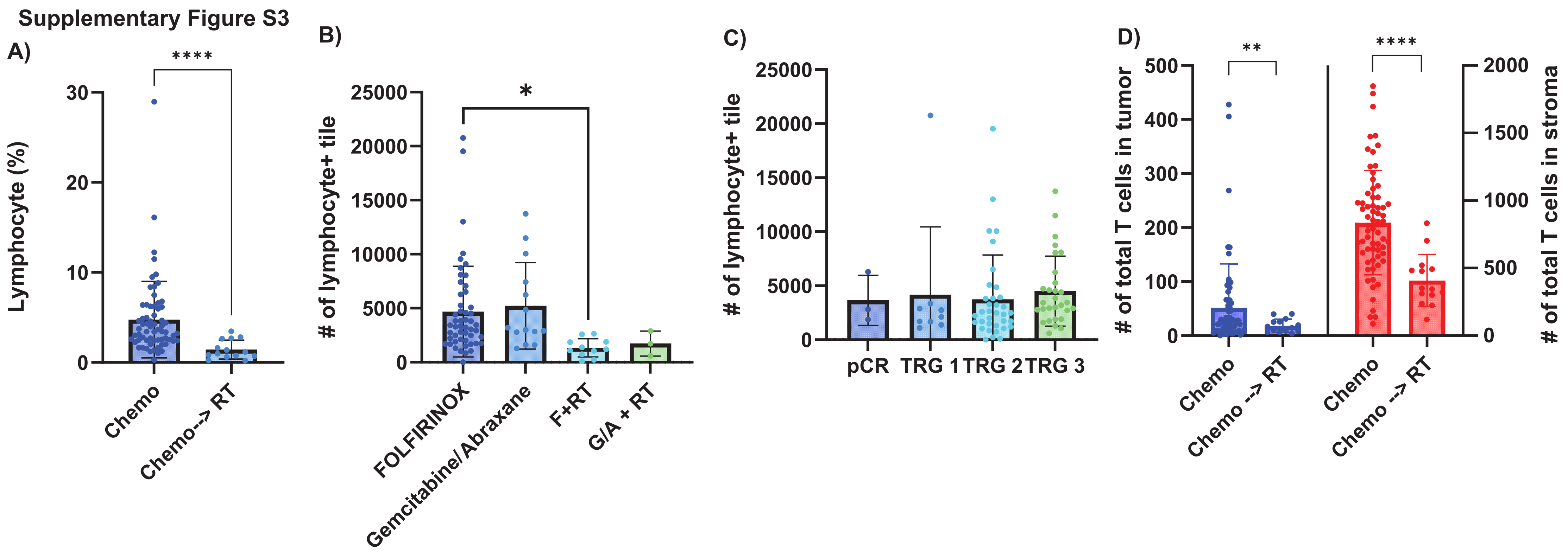

Supplementary Figure S3. Tumor infiltrating lymphocyte quantification. A) Percent of cells that are lymphocytes based on H&E analysis. B,C) Number of lymphocyte-positive tiles by neoadjuvant regimen B) and by tumor regression grade (TRG) C) using H&E analysis. pCR= pathologic complete response. D) Number of total T cells by neoadjuvant regimen based on multiplex immunohistochemistry analysis. RT= radiation. *p<0.05, **p<0.01, ****p<0.0001.
